# Impact of Intraoperative Findings on Hearing in Revision Ear Surgery

**DOI:** 10.22038/IJORL.2023.70251.3386

**Published:** 2023-05

**Authors:** Andro Kosec, Josipa Zivko, Andro Kurtic, Mihael Ries, Dejan Tomljenovic, Jakov Ajduk

**Affiliations:** 1 *Department of Otorhinolaryngology and Head and Neck Surgery, University Hospital Center Sestre Milosrdnice, Vinogradska cesta 29, Zagreb, Croatia. *; 2 *School of Medicine, University of Zagreb, Šalata 3b, Zagreb, Croatia. *

**Keywords:** Chronic otitis media, Cholesteatoma, Hearing outcomes Revision ear surgery, Tympanomastoidectomy

## Abstract

**Introduction:**

Hearing results after chronic ear surgery encompass recurrence, localization and extent of cholesteatoma, type of surgery, ossiculoplasty methods, but rarely interpret intraoperative findings. This study aimed to analyze the impact of intraoperative findings in revision tympanomastoidectomy in predicting postoperative hearing.

**Materials and Methods:**

This was a retrospective non-randomized cohort of 101 patients treated for recurrent chronic otitis media by tympanomastoidectomy. The patients’ demographics, localizations of disease recurrence and perioperative hearing results were analyzed.

**Results:**

Logistic regression showed that presence of tympanic perforation (p=0.036), ossicular chain damage (p=0.006), were negatively associated with improved hearing postoperatively. Attic cholesteatoma was associated with better postoperative hearing (p=0.045). Presence of tympanic perforation (p=0.050), alongside perifacial localization of imflammation (p=0.021) and ossicle destruction (p=0.013) were associated with worse postoperative hearing results. Multivariate analysis confirmed that tympanic perforation (p=0.040, F=4.401), and ossicular chain involvement (p=0.025, F=5.249), were consistent negative predictors of hearing improvement, while postoperative deterioration of hearing was associated with tympanic perforation (p=0.038, F=4.465) and facial nerve dehiscence (p=0.045, F=4.160).

**Conclusions:**

Comparison of postoperative revision tympanomastoidectomy hearing outcomes revealed significant positive reductions in air-bone gap values, primarily at low and mid frequencies. Postoperative hearing results at high frequencies are not affected by revision surgery.

## Introduction

Revision canal-wall-up (CWU) and canal-wall-down (CWD) tympanomastoidectomy represent strategies aimed at eliminating recurrent or residual pathological processes from the tympanic cavity and reconstructing the ossicles and tympanic membrane (1,2). 

The pattern of cholesteatoma spread is determined by the place of its origin, involving and eroding surrounding structures and causing intratemporal and intracranial complications (3,4). While multi-staged surgeries are common, the primary objective in revision surgery is always disease eradication, and hearing restoration is addressed after cholesteatoma removal (5). 

Many papers compare hearing outcomes after primary CWU and CWD procedures, listing recidivism and disease patterns, surgery type and hearing restoration methods as factors influencing postoperative hearing outcomes (6-9). However, little literature is available to identify the correlation between intraoperative findings and hearing results. Predicting short-term hearing results, definitive hearing outcomes, and associated complications has been challenging because of population heterogeneity, insufficient length of follow-up, and absent standardized outcome-related data (10). 

The aim of this study was to analyze localization of disease in revision tympanomastoidectomy procedures and link them to postoperative hearing outcomes. It is our hypothesis that localization of disease recurrence may be helpful in assessing postoperative hearing outcomes.

## Materials and Methods 

Data of 101 chronic otitis media patients that underwent tympanomastoidectomy for recurrent disease were enrolled in this retrospective comparative cohort study. The study protocol received IRB approval according to relevant ethical Helsinki principles (Approval number: 251-29-11-21-01-5). 

Inclusion criteria included the following: patients that underwent revision tympanomastoidectomy (canal wall up/down) from January 1st, 2010 to May 1st, 2019 in a high-volume otologic center, if the procedures were carried out by the same otologist, with available surgical data and preoperative and postoperative hearing outcomes recorded. Our follow-up concept was scheduling bi-monthly exams and performing planned second-look surgery in all suspect patients 6 months after the initial surgery. Non EPI-diffusion MRI was not routinely used. 

Indications for revision surgery were constant otorrhea with suspected recurring disease. Variables included demographics, type and number of surgeries, intraoperative findings, pure tone average air-bone gap (ABG) values (dB) concerning speech discriminating frequencies (500 Hz - 4000 Hz), in accordance with Committee on Hearing and Equilibrium recommendations. 

**Fig 1:**
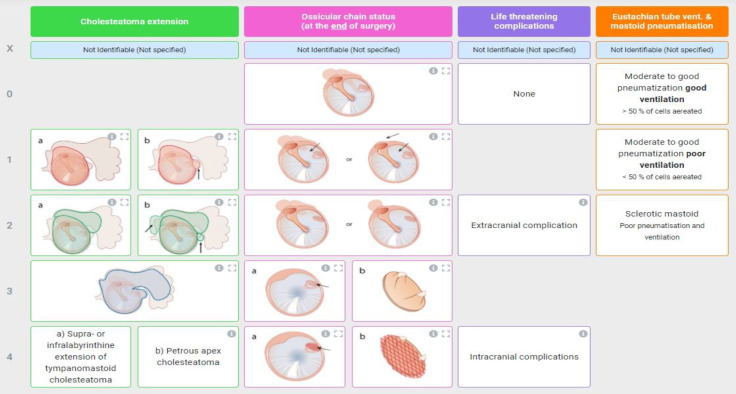
ChOLE classification; Ch – Cholesteatoma extension, O – Ossicular chain status (at the end of surgery), L – Life threatening complications, E – Eustachian tube ventilation and mastoid pneumatisation. Staging classification; Stage I – sum of classification values between 1 and 3 points, Stage II – sum of classification values between 4 and 8 points, Stage III – sum of classification values over 8 points

Average ABG was calculated from bone conduction (BC) – air conduction (AC) difference at frequencies of 500 - 4000 Hz, following American Academy of Otolaryngology, Head and Neck Surgery (AAO-HNS) recommendations, using an AC40 (Interacoustics, Middelfart, Denmark) audiometer with TDH-39 earphones (11). Informed consent was mandatory for all study participants. The extent of disease at revision surgery was noted using a ChOLE scoring system, attributing stages I-III to each of our patients with cholesteatoma depending on the extent of disease noted intraoperatively (Figure 1) (12).

Exclusion criteria were: absent surgical details, deafness and absent audiometric data. This excluded 32 patients from the initial cohort (3 deaf patients, 10 insufficient intraoperative details and 19 lacking audiometric data). In the cohort with complete follow-up (69 patients), twelve anatomical sites and extent of disease were noted, and analyzed with regard to postoperative hearing outcomes. Postoperative hearing threshold gain in decibels was labeled as the primary outcome. 

The follow-up PTA was performed 6 months after surgery. A postoperative ABG change of > 10 dB was considered to be significant. Statistics were calculated using SPSS (Version 22.0 © 2013. Armonk, NY: IBM Corp). Associations between variables were analyzed using binary logistic regression and a general linear model multivariate analysis of variance (MANOVA). All tests of were performed using a two-sided 5% type I error rate.

## Results

**Preoperative findings**

Mean age of patients was 39,91 years, from 7 to 81 years. The study included 33 male and 36 female patients. There were 41 patients with defects in the tympanic membrane, while 33 had suspected cholesteatoma on otoscopy. 

Out of 69 patients, 39 were surgically treated only once, while 30 had multiple procedures, with 1,47 surgeries per patient on average, and average time since last surgery was 8,44 years. Of the 69 patients in the study, 13 had been previously treated in our otologic center. There were 47 CWU tympanomastoidectomies and 22 CWD tympanomastoidectomies. In the CWD group, seven patients were previously treated by a CWU surgery, and 15 had disease that required a CWD surgery outright. 

***Intraoperative findings and hearing outcomes***

The tympanic membrane was perforated in 59,42% cases, and was affected by cholesteatoma-related retraction pockets in approximately half of the patients. Recurrent disease and inflammation were the ossicles in 55,07%. 

**Fig 2:**
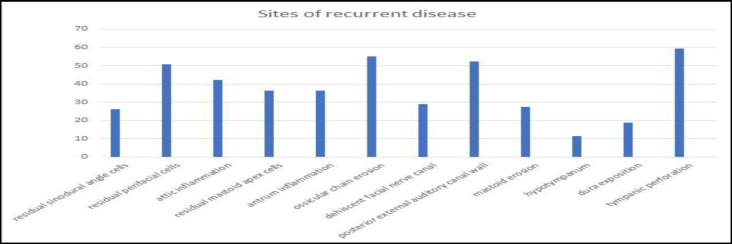
Sites of recurrent disease noted intraoperatively

This area was followed by erosion of the posterior and superior external auditory canal wall in 52,17% patients, high facial ridge in 50,72% and attic recurrent disease in 42,03%, mastoid recurrence in 36,23% and 36,23% patients (Figure 2).

Cholesteatoma recurred in 33 (47,83%) patients. According to the ChOLE classification in patients with cholesteatoma, there were 18 patients in stage I (54,5%), twelve patients in stage II (36,4%) and three 

patients in stage III (9,1%).

The average preoperative and postoperative PTA values and postoperative ABG shifts were analyzed at 500 Hz, - 4000 Hz frequencies, depending on surgery type (Table 1). 

**Table 1:** The average preoperative and postoperative PTA values and postoperative ABG shifts affecting 500 Hz - 4000 Hz frequencies depending on surgery type

	***CWU***	***CWD***
*Preop 500 Hz*	41,17 ± 20,67	54,77 ± 28,26
*Postop 500 Hz*	38,04 ± 20,69	60,00 ± 23,28
*Preop 1000 Hz*	39,26 ±19,39	53,86 ± 31,96
*Postop 1000 Hz*	36,06 ± 18,76	57,95 ± 26,03
*Preop 2000 Hz*	36,06 ± 20,08	54,77 ± 33,96
*Postop 2000 Hz*	34,47 ± 20,44	60,45 ± 29,84
*Preop 4000 Hz*	50,11 ± 24,77	70,45 ± 32,11
*Postop 4000 Hz*	50,11 ± 27,18	71,36 ± 26,91
*ABG 500 Hz*	2,23 ±12,11	-5,23 ±19,28
*ABG 1000 Hz*	3,19±11,41	-4,09±20,76
*ABG 2000 Hz*	1,60±13,49	-5,68±19,96
*ABG 4000 Hz*	0±12,42	-0,91±15,57
*ABG total*	6,17 ± 37,26	-14,32 ± 70,18

The average ABG reduction at 500 Hz was 9.06 dB (±11.94), at 1000 Hz it was 11.41 dB (±9,179), at 2000 Hz it was 8.28 dB (±13.051), and at 4000 Hz, it was 8.28 dB (±9,035). The average total postoperative reduction of the ABG across all speech discriminating frequencies was 37.34 dB (±26.089).

Logistic regression dependent variables were twofold - improvement of postoperative hearing (reduction of air-bone gap) and deterioration of postoperative hearing (increase of air-bone gap) (Tables 2-6).

**Table 2:** Intraoperative sites linked to hearing improvement and deterioration (binary logistic regression). For other sites p>0,05

	***Hearing improvement***			***Hearing deterioration***			
***Intraoperative site***	p	OR	CI	**Intraoperative site**	p	OR	CI
*Tympanic perforation*	0,036	0,289	0,090-0,922	Tympanic perforation	0,050	3,335	0,967-11,503
*Ossicular chain*	0,006	0,182	0,054-0,610	Ossicular chain	0,013	6,612	1,195-36,582
*Attic*	0,045	3,964	1,032-15,225	Facial ridge	0,021	6,512	1,131-37,502

**Table 3:** Intraoperative sites linked to hearing improvement and deterioration (multivariate analysis). For other sites p>0,05

	***Hearing improvement***			***Hearing deterioration***			
*Intraoperative site*	p	F		Intraoperative site	p	F	
*Tympanic perforation*	0,040	4.401		Tympanic perforation	0,038	4.465	
*Ossicular chain*	0,025	5.249		Facial nerve dehiscence	0,045	4.160	

**Table 4:** Impact of surgery type on hearing outcome

***PTA***	***OR***	***p***	***ABG***	***OR***	***p***
*Preoperative 0,5 kHz*	4,880	0,027	ABG 0,5 kHz	3,621	0,057
*Postoperative 0,5 kHz*	12,170	0,000	ABG 1 kHz	3,344	0,067
*Preoperative 1 kHz*	5,261	0,022	ABG 2 kHz	3,023	0,082
*Postoperative 1 kHz*	13,174	0,000	ABG 4 kHz	0,068	0,794
*Preoperative 2 kHz*	7,538	0,006	ABG total	2,412	0,120
*Postoperative 2 kHz*	14,539	0,000			
*Preoperative 4 kHz*	7,635	0,006			
*Postoperative 4 kHz*	8,350	0,004			

**Table 5:** Impact of cholesteatoma on hearing

***Frequency***	***OR***	***p***
*Preoperative 0,5 kHz*	0,987	0,320
*Postoperative 0,5 kHz*	4,621	0,032
*Preoperative 1 kHz*	0,480	0,488
*Postoperative 1 kHz*	3,829	0,050
*Preoperative 2 kHz*	1,829	0,176
*Postoperative 2 kHz*	2,248	0,134
*Preoperative 4 kHz*	3,860	0,049
*Postoperative 4 kHz*	3,882	0,049

**Table 6:** *Frequency dependent hearing improvement*

*Frequency*	*OR*	*p*
*Preoperative 0,5 kHz*	0,417	0,518
*Postoperative 0,5 kHz*	5,686	0,017
*Preoperative 1 kHz*	1,615	0,204
*Postoperative 1 kHz*	4,598	0,032
*Preoperative 2 kHz*	0,679	0,410
*Postoperative 2 kHz*	3,286	0,070
*Preoperative 4 kHz*	0,568	0,451
*Postoperative 4 kHz*	2,467	0,116

The logistic regression model did not identify ChOLE stage as a predictor of hearing outcome. However, when individual anatomical sites of residual disease and cholesteatoma were compared, the presence of tympanic perforation (p=0.036, OR 0.289, CI 0.090-0.922), and ossicular chain involvement (p=0.006, OR 0.182, CI 0.054-0.610), were identified as the strongest negative predictors of hearing improvement (air-bone gap reduction). Attic involvement was identified as a positive predictor of hearing improvement (air-bone gap reduction) (p=0.045, OR 3.9644, CI 1.032-15.225). When analyzing intraoperative sites at risk for the deterioration of hearing (increased air-bone gap), tympanic perforation (p=0.050, OR 3.335, CI 0.967-11.503), perifacial residual disease (p=0.021, OR 6.512, CI 1.131-37.502) and ossicular chain involvement (p=0.013, OR 6.612, CI 1.195-36.582) were singled out as the most accurate predictor variables of hearing deterioration.

In order to control for potential sources of bias and confirm our results, a multivariate analysis using a general linear model multivariate analysis of variance (MANOVA) was used. It confirmed that tympanic perforation (p=0.040, F=4.401), and ossicular chain involvement (p=0.025, F=5.249), were consistent negative predictors of hearing improvement. 

When analyzing factors correlated with postoperative deterioration of hearing, tympanic perforation (p=0.038, F=4.465) and facial nerve dehiscence (p=0.045, F=4.160) were the strongest predictors of hearing deterioration postoperatively.

After analyzing the regression model for correlation of intraoperative sites, PTA hearing thresholds and the type of revision surgery, CWU patients had a significantly lower postoperative PTA values in all frequencies (p=0.003, OR 8.635), but no postoperative ABG reduction was identified among the CWU and CWD patients. In contrast, the intraoperative presence of recurring cholesteatoma was correlated with a lower postoperative PTA hearing threshold affecting 500 Hz, 1000 Hz, but not the 2000 Hz and 4000 Hz frequencies in both CWU and CWD surgeries (p=0.032, OR 4.621). In patients that demonstrated a postoperative ABG reduction >10 dB, it was statistically significantly demonstrated in the 500 Hz (p=0.017, OR 5.686) and 1000 Hz frequencies (p=0.032, OR 4.598), while the 2000 Hz and 4000 Hz frequencies remained unchanged postoperatively. 

## Discussion

Both canal-wall up and canal-wall down techniques represent two strategies for chronic otitis media management. Literature has discussed the advantages and disadvantages of these techniques extensively (13-15). We analyzed the sites of disease recurrence in order to single out the most common localizations associated with previous surgical failure. The sinodural angle and high facial ridge, also known as “the beginner’s ridge”, are frequently emphasized as most common sites of recurrence(16-18). Common areas of residual disease in our patients were the ossicles, posterosuperior external auditory canal and facial ridge cells. 

Cholesteatoma was found in 47,83% revision cases, forming a retraction pocket (recurrent disease) or left behind after the previous surgery (residual disease). If the patient has undergone CWU previously, a more radical CWD procedure may be required in order to eradicate the disease (15). The presence of cholesteatoma was linked to poor hearing thresholds at low frequencies (500 and 1000 Hz) after revision surgery in our cohort. One would expect that extent of the disease affects audiologic outcome, complication rate, and recidivism, but our data set did not show correlation of ChOLE cholesteatoma-attributed stage and hearing outcome. However, after analyzing frequency-specific correlations, important results were found because the pattern of postoperative hearing perception may differ, even though the pure tone thresholds remain unchanged. In addition, they cannot be uniformly attributed to a certain cholesteatoma stage (13,19).

Associating recurrence sites and hearing outcomes has significant prognostic value. Conductive hearing loss following chronic otitis media surgery can be anticipated by analyzing ossicular and acoustic coupling (19,20). Postoperative hearing improvement in our cohort was affected by recurrence sites involving the ossicles, tympanic reperforation (negative predictors) and attic (positive predictor), with ossicular chain involvement and tympanic reperforation identified as significant in the multivariate analysis as well. Attic cholesteatoma is formed from the retraction pocket of the flaccid part of the tympanic membrane (Schrapnell’s membrane) and develops in Prussak’s space. Gulustan et al. state that the bone conduction threshold is lower if cholesteatoma occurs in the attic, comparing to other sites (21). Stankovic et al. noted a significant ABG reduction in a patient group with attic cholesteatoma (22). A potential cause of this observation is the fact that cholesteatoma, while developing in the attic, avoids incudostapedial joint destruction which is located in mesotympanon and is considered to be the most vulnerable spot in the tympanic cavity. Furthermore, cholesteatoma which originates from the flaccid part causes the osteolysis of the ossicles in a lesser percentage (75%) comparing to cholesteatoma arising from the pars tensa (90%) (23,24).

Some localizations of the recurrent/residual disease are associated with hearing improvement, while others with hearing deterioration, indicating that likely and probable postoperative hearing outcomes have to be discussed in a realistic and data-reliant way (24).

Positive predictors of hearing deterioration in our study were tympanic perforation, perifacial residual disease and involvement of the ossicles, all contributing to defective sound transmission, with tympanic perforation and facial nerve canal dehiscence confirmed as significant predictors by multivariate analysis. Facial nerve dehiscence is a frequent anatomical landmark in all patients, especially in those with longstanding disease, and may be associated with adjoining difficulties in ossicular defect reconstruction in recurring disease and revision surgery (24,25)

A tympanic membrane reperforation was found in 59,42 % cases. It causes persistent otorrhea and conductive hearing loss and those are symptoms that reduce the quality of life and represents an important etiological factor in cholesteatoma formation since squamous epithelial cells can migrate from the external ear to the tympanic cavity. Recurrent perforation and ossicle involvement were identified as the most important negative predictors of hearing improvement. When comparing the outcomes of primary tympanoplasties with revision cases with ossicular destruction, primary surgeries provide better results, both with regard to closing the perforation of the tympanic membrane and hearing improvement (19). In our cohort, when analyzing only patients with tympanic perforation and postoperative ABG reduction, a significant improvement was observed in the 2000 and 4000 Hz range, consistent with the known fact that no technique used is particularly useful in restoring hearing in the > 4000 Hz range. In 9 of our patients (13%), a postoperative worsening of ABG in the 4000 Hz range and above was noted. This corresponds to known data, especially prone to occur in younger patients with advanced cholesteatoma (26).

There are many factors affecting the choice of the adequate surgery technique. CWD procedure provides better visualization and the complete removal of cholesteatoma. Kerckhoff et al. claim that the risk of cholesteatoma recurrence and residual cholesteatoma is lower in CWD procedures (0-13,2%) comparing to CWU (16,7-61%) (23,25). However, after a CWD procedure there is a collection of keratin debris in the patient’s ear and it becomes prone to infection after contact with water (27). More authors argue that better hearing outcomes are achieved using the CWU procedure in contrast to CWD (10,11,16,17). Contrarily, some authors claim there is no significant difference, particularly after a long period of follow-up (13,14,27,28). It is necessary to have in mind that besides the type of surgery, there are other factors affecting hearing results, such as the function of the Eustachian tube, tympanic cavity granulation and infection. 

Our data show that the majority of patients with cholesteatoma have poor preoperative hearing (ABG >20 dB). Patients who have had CWU surgery showed significantly lower preoperative and postoperative PTA values at all frequencies (p=0,003, OR 8,635), with no difference in postoperative ABG reduction was noted among the CWU and CWD groups, confirming that both types of surgeries are suitable for achieving hearing improvement, which is not significantly different in terms of total ABG reduction, but with inferior preoperative hearing thresholds in CWD patients. 

In patients that demonstrated a significant postoperative ABG reduction, it mostly involved 500 Hz and 1000 Hz frequencies, which is consistent to previous studies (16). In normal ear physiology, the tympanic membrane and ossicles function as a compound lever and it increases the pressure which is transferred to the inner ear during sound transmission. Pressure amplification is essential to convey mechanical motion from the air to the inner ear fluid. This pressure gain is frequency-dependent, especially at 1 kHz, with mean peak gain at 26,6 dB. At higher frequencies it decreases by -8,6 dB per every octave. At 4 000 Hz, which is the highest analyzed frequency in this paper, pressure gain is only 6,5 dB. This mechanism is disturbed by middle ear diseases. After reconstructing both the tympanic membrane and ossicles, we can improve hearing at frequencies which are tightly related to the function of these structures and those are low and mid frequencies (19,22). Poor hearing outcomes at higher frequencies can also be linked to previous disease, aging and iatrogenic related damage to the stapes, *fenestra rotunda* and cochlear promontory, which is crucial for the perception of high frequency sounds (22,26). No differences were seen between children and adults in our results regarding reports of pediatric cholesteatoma being more aggressive than disease acquired in adulthood (29). The canal-wall up surgical strategy is often impossible in revision surgery, while the canal-wall down strategy enables superior anatomical visualization, and inferior, but still functional postoperative hearing results. 

The main limitations of this study are a small, heterogeneous sample, retrospective data collection and a short postoperative follow-up interval. Hearing results had precedence over reconstructive materials or techniques in our analysis. Analyzing hearing outcomes between CWU and CWD procedures might pose a risk of bias, due to differences in operating technique, indication and extent of surgery.

## Conclusion

In revision chronic otitis media surgery, the type of procedure can significantly affect postoperative hearing. Air-bone-gap shifts indicated that improvements are possible, primarily at low and middle frequencies, with a sustained hearing loss occuring at high frequencies. Sites of recurrence of the disease can be linked to the postoperative hearing outcome which is helpful in determining the prognosis. Careful surgical planning using extrapolated data on anatomical pitfalls is important to reduce the need for further revision surgeries.
